# AvBD1 nucleotide polymorphisms, peptide antimicrobial activities and microbial colonisation of the broiler chicken gut

**DOI:** 10.1186/s12864-017-4034-6

**Published:** 2017-08-18

**Authors:** Kevin Cadwell, Sherko S. Niranji, Vanessa L. Armstrong, Catherine A. Mowbray, Richard Bailey, Kellie A Watson, Judith Hall

**Affiliations:** 1 Institute for Cell and Molecular Biosciences, Newcastle University, Newcastle upon Tyne, NE2 4HH UK; 2 Aviagen Ltd, Newbridge, Midlothian, EU28 8SZ UK; 3 Present address: School of Biomedical Sciences, Newcastle University, Newcastle upon Tyne, UK; 4 Present address: College of Veterinary Medicine, University of Sulaimani, Sulaymaniyah, Iraq

**Keywords:** Avian β-defensin 1, Gene SNPs, Antimicrobial activity, Membrane permeabilisation, Gut bacteria

## Abstract

**Background:**

The importance of poultry as a global source of protein underpins the chicken genome and associated SNP data as key tools in selecting and breeding healthy robust birds with improved disease resistance. SNPs affecting host peptides involved in the innate defences tend to be rare, but three non-synonymous SNPs in the avian β-defensin (AvBD1) gene encoding the variant peptides NYH, SSY and NYY were identified that segregated specifically to three lines of commercial broiler chickens Line X (LX), Line Y(LY) and Line Z. The impacts of such amino acid changes on peptide antimicrobial properties were analysed in vitro and described in relation to the caecal microbiota and gut health of LX and LY birds.

**Results:**

Time-kill and radial immune diffusion assays indicated all three peptides to have antimicrobial properties against gram negative and positive bacteria with a hierarchy of NYH > SSY > NYY. Calcein leakage assays supported AvBD1 NYH as the most potent membrane permeabilising agent although no significant differences in secondary structure were identified to explain this. However, distinct claw regions, identified by 3D modelling and proposed to play a key role in microbial membrane attachment, and permeation, were more distinct in the NYH model. In vivo AvBD1 synthesis was detected in the bird gut epithelia. Analyses of the caecal gut microbiota of young day 4 birds suggested trends in *Lactobacilli sp.* colonisation at days 4 (9% LX vs × 30% LY) and 28 (20% LX vs 12% LY) respectively, but these were not statistically significant (*P* > 0.05).

**Conclusion:**

Amino acid changes altering the killing capacity of the AvBD1 peptide were associated with two different bird lines, but such changes did not impact significantly on caecal gut microbiota.

## Background

The defensins comprise groups of innate effectors synthesised by epithelia and antigen presenting cells that function as a first line defence mechanism to protect the host from infection. These small molecular weight molecules, less than 50 aa in length and 2 to 6 kDa in size, are noted for their broad-spectrum anti-microbial properties, which are facilitated through their small size, cationic charge and preponderance of hydrophobic amino acids [1, 2]. In addition to their killing activities mammalian defensins exhibit an array of immuno-protective functions that include wound healing, chemotaxis and mast cell degranulation, while roles in development and reproduction have also been reported [3]. While mammals are characterised by three defensin families namely α, β, and θ, with the latter being confined to macaques and baboons, birds synthesise only one family, the β-defensins. Evolutionarily these molecules represent the oldest of the defensin families, are found in most classes of vertebrates and typified by the C1-C5, C2-C4 and C3-C6 disulphide bridging pattern of the six conserved cysteines. In the chicken genome the family includes a cluster of 14 distinct β-defensin (AvBD) genes located within 86 kb of chromosome 3 that has evolved, presumably, through gene duplication [4, 5].

As well as roles in the host innate defences, which pivotally involve protecting the epithelia from microbial assault, the defensins also function in regulating the endogenous gut microflora [6]. Thus any mutations that affect peptide expression and/or structure, and hence microbial killing properties, can potentially lead to an altered innate immunity and/or microbiota, resulting in an increased susceptibility of the host to disease. As a consequence, natural allelic variations or SNPs within the defensin loci are uncommon and are generally identified through the increased sensitivity of individuals to diseases, particularly those involving epithelial-microbial interactions [7].

Poultry, particularly chicken, are a major source of protein worldwide and the commercial, and nutritional value of the meat has for decades, underpinned research into optimising bird production. Recent research has also focussed on rearing ‘robust’ healthy birds that not only show optimal feed to gain ratios, but can also resist infectious and/or zoonotic diseases [8]. Genomic and SNP data has therefore become of increasing importance to breeders, and SNPs identified within the defensin genes have already been proposed as molecular markers to facilitate the selection of commercial poultry resistant to enteric pathogens [9]. Yet, while many SNPs associate with the required trait in multiple rearing environments a number have been identified that are particularly sensitive to the external conditions. For example, the *TGFβ3 Msp1* SNP associates with increased mortality in high, but not low hygiene environments. These observations suggest that interactions between genetic and environmental factors are not only important, but multifaceted, and for breeding purposes direct the selection of SNPs less sensitive to environmental changes [10].

Non-synonomous amino acid changes that affect peptide functionality can impact on an organism’s phenotype although for molecules functioning in innate immunity, genetic changes that direct such mutations tend to be infrequent. However, in two domestic chicken breeds, White Leghorn and Cornish, the chicken NK-lysin gene, encoding an innate defence molecule with microbiocidal and cytolytic activities, has been shown to carry coding sequence variation [11]. The polymorphism involves a single nonsynonymous SNP at nucleotide 271 that results in an Asn (N) to Asp (D) amino acid substitution and peptides synthesised to model this change indicate the N29 N peptide to exhibit greater antibacterial activity compared to the N29D variant. Within an array of inbred and heritage chicken breeds the A SNP encoding an asparagine (N) dominates, suggesting that positive selection of this amino acid has been driven by the superior killing properties of the peptide in response to the global microbial challenge [12].

To date there are limited reports describing natural allelic variation of the AvBDs and the consequences thereof. In this study analyses of SNP panels from different commercial elite broiler chicken lines identified three non-synonymous SNPs in the AvBD1 mature peptide coding region [13]. The SNPs resulted in amino acid changes at positions 10, 20 and 32 with the variant peptides identified as NYH, SSY and S/NYY. The ‘NYH’ peptide represented the major AvBD1 form synthesized in Line X (LX) birds, ‘SSY’ typified AvBD1 in Line Y (LY) birds, while ‘N/SYY’ represented the peptides synthesised by Line Z (LZ). To explore the impact of the AvBD1 SNPs this study, utilising synthetic peptides modelling AvBD1 NYH, SSY and NYY, examined and compared the effects of the amino acid changes on the antimicrobial properties of the peptides. These data were discussed in relation to the gut microbiotae of LX and LY birds.

## Methods

### Bacterial growth

Bacteria used in the study were isolated from the gastrointestinal tracts of birds reared on commercial farms. The *Escherichia coli*, *Barnsiella viscericola, Bacteroides dorei and Lactobacillus johnsonii* strains were isolated post mortem from a Ross 308 broiler chicken. The *Enterococcus faecalis* isolate was similarly isolated, but from a different bird. Blood agar was used for the growth of *E. coli* and *E. faecalis*; brain heart infusion (BHI) broth and agar were used for the growth of *B. viscericola.* Rogosa and Sharpe (MRS) broth and agar were used for culturing *L. johnsonni. B. dorei* strains were grown in Tryptone Yeast Glucose (TYG) broth containing tryptone peptone (1 g), bacto yeast extract (0.5 g), glucose (0.2 g), cysteine (free base) (0.05 g), 1 M KPO_4_ pH 7.2 (10 ml), Vitamin K solution, 1 mg/ml (1 ml), TYG salts (4 ml), 0.8% CaCl_2_ (0.1 ml), FeSO_4_, 0.4 mg/ml (0.1 ml), resazurin, 0.25 mg/ml (0.4 ml) and H_2_O (85 ml). Prior to culturing, haematin (*w*/*v* %) was added to a final concentration of 0.1% *w*/*v*. *E. coli* and *E. faecalis* were grown aerobically while *B. dorei*, *L. johnsonii* and *B. viscericola* were cultivated on plates contained in an anaerobic jar (Anaerocult® system, VWR International, U.K.).

### Antimicrobial assays (AMA)

#### Time-kill assay

A single colony of either *E. coli* or *E. faecalis* was grown overnight in 5 ml broth at 37 °C with gentle shaking. 10 ml LB containing 100 mM glucose was inoculated with 200 μl of the overnight culture and the bacteria grown to mid-log (OD_600nm_ 0.3–0.6). Working bacterial stocks were prepared by diluting the culture 1:1000 with 0.1 M phosphate buffered saline (PBS). The colony counting assay [14] was modified to use 96-well microtitre plates and 2 h incubation time periods. The bacterial growth inhibited by each peptide sample was presented relative to the PBS control, which was assigned a growth value of 100%. Each assay was repeated on at least three separate occasions with minimum of two replicates for each peptide/control sample.

#### Radial diffusion assay

Assays were performed as described for human β-defensin-1 [15]. Briefly bacteria were grown to mid-log from an overnight culture and pelleted by centrifugation (1000 g) for 10 min. The media was removed, the pellet was washed with cold 10 mM phosphate buffer, re-centrifuged, re-suspended to an OD_600nm_ of 0.1 nm (against a buffer control) and kept on ice until required. 150 μl of diluted bacterial suspension (OD_600nm_ 0.1 nm) was added to a 10 ml aliquot of liquid ‘underlay’ gel at 45 °C, gently mixed, poured into a petri-dish and allowed to set. Wells (3 mm) were punched into the gel and either peptide or PBS buffer added. After 3 h, 5 ml of melted overlay was added to each plate, allowed to dry and the plates incubated overnight. Zones of bacterial inhibition were photographed and measured using ImageJ software (imagej.nih.gov/ij/, National Institutes of Health, Maryland, U.S.A.).

For the assessment of peptide activities against anaerobic bacteria (*B. dorei*, *B. viscericola* and *L. johnsonii*) the underlay and overlay aliquots also contained a reducing agent, dithiothreitol (DTT) (1 mM), and a redox-indicator, resazurine (1 μg/ml). Anaerobic bacteria were cultured under anaerobic conditions using the Anaerocult® system (VWR International, U.K.).

### Synthetic (s) AVBD1 peptides

Three AvBD1 variants termed ‘NYH’, ‘SSY’ and ‘NYY’ based on their primary sequence were synthesised by PeptideSynthetics (Hampshire, UK) with >95% purity. Lyophilised peptide was stored at −20 °C and a working stock of 1 mg/ml prepared by dissolving 1 mg peptide in 20 μl of 10% acetic acid and the volume increased to 1 ml using Milli-Q water.

### CD spectroscopy

CD spectroscopy was carried out on a Jasco-810 CD spectropolarimeter. For far-UV measurements (250–185 nm) at room temperature 0.2 cm Hellma stoppered cuvettes were used, the bandwidth was set to 0.2 nm, data pitch 0.5 nm and scanning speed 100 nm/min. Secondary structures were predicted using Dichroweb software (http://dichroweb.cryst.bbk.ac.uk/html/home.shtml). The CD spectra data were inputted and outputted as text files and delta epsilon units from 190 to 240 nm using K2D software [16].

### Calcein leakage assay

Lipid stock solution (50 mg/ml) was prepared by mixing soya bean, type II S Phospholipid powder, L-α- Phosphatidylcholine (Sigma) with hexane (BHD, VWR, UK) and few drops of ethanol, and stored at −20 °C. For experiments, 2 ml (100 mg) of the lipid solution were dried to a film in a round bottom flask. Calcein solution (20 mM) was prepared by dissolving calcein (Sigma) in 100 mM sodium phosphate buffer and filtering through a 0.2 μm membrane. To create calcein-trapped multilamelar (MLV) vesicles, 2 ml of the calcein solution was added into the lipid film and this shaken with clean glass beds (2.5–3.5 mm) for 20 min at room temperature. Unilamellar vesicles (ULV) were prepared by passing the MLV through a miniextruder (Avanti) with 100 nm polycarbonated membrane at least 11 times. Untrapped (free) calcein molecules were removed using a PD-10 desalting column (GE Healthcare). Calcein leakage from the ULV liposomes was monitored using a fluorescent spectrophotometer (Varian) and Cary Eclipse software, Excitation (493 nm) and emission (505-600 nm). TritonX-100 (1% final concentration) was used as a 100% leakage positive control. Leakage was calculated using the equation: Leakage % = (Fp-F0)/ (Ft-F0) *100%, when Fp is the measurement of fluorescent leakage by the peptides, Ft is the complete leakage by TritonX and F0 is intact vesicles before adding peptide or TritonX.

### Modelling

The AvBD1 peptide structures were modelled using Raptor X online software (http://raptorx.uchicago.edu/) [17]. The three dimensional structure prediction used the Penguin AvBD 103b (Spheniscin-2) structure, solved previously using two dimensional NMR, as its template [18].

### SNP genotyping

SNP genotyping data related to ten commercial broiler breeding lines, including LX, LY and LZ, and a minimum of 200 birds per line [19]. Genotyping and genotype scoring was performed by Illumina using a 12 K SNP array panel. Pooled genomic DNA was also directly sequenced (GENEVISION, Newcastle upon Tyne) and SNP allele frequency determined [20]. Each pool contained DNA from 20 birds; six pools were sequenced for LX and LY, and five pools for LZ.

### Birds & Rearing Environment

Two independent trials were completed each involving thirty newly hatched birds from the two Aviagen broiler chicken genotypes LX and LY. In trial 1, 15 LX and 15 LY birds, were assigned to pens each containing of 5 birds within a ‘sib-test’ facility [21]. All birds were fed standard feed ration (maize crumble) and at 4, 21 and 28 days the gut health of each bird was assessed and scored for redness, watery digesta and gut tone. Each gut was assigned a score of 0 (normal), 1 (some abnormalities) or 2 (very abnormal) with poorest guts scoring 6 across all three gut-health traits. This procedure was repeated for trial 2.

### Gut microbiome

DNA extraction of pooled caecal digesta samples (maximum of 5 birds/pool) was performed using the DNAzol™ kit (Life Technologies Ltd., Paisley, UK). DNA pellets were re-suspended in 200 μl of TE elution buffer (Qiagen, UK) with concentration and purity assessed by Nanodrop. Bacterial DNA was analysed by 454-pyro sequencing (Roche, Indianapolis, U.S.A) targeting the V4-V5 region of the bacterial 16S ribosomal gene as a service by the Animal Health and Veterinary Laboratories Agency (AHVL), Weybridge, U.K. Sequencing returned over 10,000 reads per sample, each read was 400–500 bp in length and any poor quality reads were discarded. Methods used for prediction of identities from the sequencing data were automated and involved using BLASTn against Ribosomal databases [22]. Data were formatted by AHVL to show percentage abundance of each identified genus in relation to the overall microbiome.

### Immunohistochemistry

A rabbit polyclonal antibody to AvBD1 was produced by Cambridge Research Biochemicals (Cleveland, U.K.) using the unique peptide antigen, GRKSDSFRKNGFC-amide and used in the IHC analyses. Avian tissue was harvested and fixed in 4% buffered formalin and subsequently stored in 70% ethanol before being processed into paraffin blocks. Tissue was sectioned at a thickness of 4 μm onto SuperFrost Plus slides (Fisher Scientific, Loughborough, U.K.) and allowed to dry for 24 to 48 h. For staining, slides were de-waxed in xylene, rehydrated through graded alcohols to water and subjected to a hydrogen peroxide block (1.5% in water) for 10 min. Following antigen retrieval by pressure cooking with EDTA (pH 8.0), staining was carried out using the Vectastain Elite ABC peroxidase kit (rabbit) (Vector Laboratories, Peterborough, U.K.) as per manufacturers’ instructions. Antibody was used at 1:250 dilution in TBS (pH 7.6) for 1 h at room temperature. The reaction was developed using the peroxidase chromogen DAB (3,3-diaminobenzedine tetrahydrochloride) as per manufacturer’s instructions and the nuclei counterstained using Mayer’s Haematoxylin and Scot’s tapwater substitute.

### Statistical analyses

Statistical analyses were performed using the Prism 5 Software package (GraphPad Software Inc., La Jolla, California, USA). For analyses of data involving more than two groups a two–way analysis of variance followed by Tukey’s multiple comparison or Bonferroni post-tests, as appropriate, was used. Significance of microbiota data between LX and LY groups at each time-point was determined by unpaired two-tailed Student’s t test. The significance level was set at 5% (*P* < 0.05).

## Results

### AvBD1 gene SNP identification

Two methods were used to identify amino acid substitutions within AvBD1, an Illumina 12 K SNP panel array, which generated Table 1 and direct sequencing that generated Table 2. Examination of the AvBD locus on chromosome 3 identified 15 SNPs that were exhibited by three bird lines LX, LY and LZ shown by linkage disequilibrium to be phylogenetically distinct [19]. Of the SNPs identified, 13 were located in the intronic and/or non-coding sequence of the AvBD genes 1 to 10 (Table 1), while the SNPs identified within the exon 1 region of AvBD3 and the exon 2 gene sequence of AvBD1 (Rs 15,457,749) were both predicted to encode non-synonomous amino acid changes. Direct sequencing of pooled DNA samples from these bird lines confirmed the frequency of the AvBD1 Rs 15,457,749 SNP Illumina data (Table 2), and identified two additional SNPs in the AvBD1 mature peptide coding region at positions c.169 (Rs 15,457,747) and c.104 (SNPAvBD1a). All three AvBD1 SNPs were located in exon 2 and encoded non-synonymous amino acid changes (Table 1). Analyses of the primary amino acid sequences of the AvBD1 variants showed that they differed in amino acids 10, 20 and 32 with NYH, SSY and S/NYY variants composed of asparagine (N)10, tyrosine (Y)20 and histidine(H)32; S10, S20 and Y32 and S/N10, Y20 and Y32, respectively. Hence, the sole variant in LX has an amino acid sequence N(10)-Y(20)-H(32), denoted NYH; the main variant in Line LY has sequence SSY and the main variant in Line Z has sequence N/SYY.

**Table 1:** AvBD SNPs. AvBD SNPs identified in commercial broiler chicken lines LX, LY and LZ using 12 K SNP panel array. Pooled genomic DNAs from 120 LX and LY birds, and up to 100 LZ birds were analysed for AvBD1 Exon 2 coding SNPs. SNP AvBD1a (A > G) at c.104, is defined as position of SNP where 1 is A of ATG start codon of AvBD1 cDNA sequence; Rs15457749 (A > C) at c.134 and Rs15457747 (C > T) at c.169. A > G represents nucleotide substitution in sense DNA strand. Data presented as mean frequency ± SEM

AvBD Gene	SNP Code		Location	LX	LY	LZ
				Frequency		
1	Rs15457749	G/T	Exon 2	0.87	0.02	0.55
1	Rs15457745	C/T	Intron	0.87	0.22	0.72
3	SNPAvBD3a	C/T	Exon 1	0.87	0.23	0.89
4	Rs16341536	T/C	5’UTR	0.99	1	0.58
6	Rs13526000	T/G	Intron	0.49	0	0.28
6	Rs16341514	G/T	Intron	0.17	1	0.28
8	Rs15457650	G/T	Intron	0.08	0	0.26
8	Rs15457653	T/C	Intron	0.58	0.21	0.72
8	SNPAvBD8a	C/T	Intron	1	1	0.82
9	Rs3137928	T/C	3’UTR	0.08	0.14	0.61
9	Rs14411786	C/T	Intron	0.97	0.86	0.65
9	SNPAvBD9a	T/C	Intron	0.99	0.86	0.36
10	Rs15457607	T/C	Intron	0.88	0.98	0.45
10	Rs14411785	T/C	5’UTR	0.94	0.86	0.65
10	SNPAvBD10a	T/C	Intron	0.85	0.98	0.44

**Table 2:** AVBD1 SNP frequencies within pooled genomic DNA samples. Encoded amino acids are presented using the one letter code system with N:Asparagine; S:Serine; Y:Tyrosine; H:Histidine﻿. Only the most common amino acid combinations of the AvBD1 peptides synthesised by LX, LY and LZ birds are presented

AvBD1 SNP	Bird Line	T base %	G base %	C Base %	±SEM	Amino acid
AvBD1a c.104 (A > G)	X (120)	100		0	0	N (A**A**T)
	Y (120)	10.7		89.3	6.6	S (A**G**T)
	Z (80)	48.8		51.2	1.2	N/S
Rs15457749 c.134 (A > C)	X (120)	100	0		0	Y (T**A**C)
	Y (120)	4.3	95.7		4.3	S (T**C**C)
	Z (100)	38.9	61.1		1.1	Y
Rs15457747 c.169 (C > T)	X (120)	13.8		86.2	3.2	H (**C**AC)
	Y (120)	89.7		10.3	7.4	Y (**T**AC)
	Z (100)	61.4		38.6	1.6	Y

### Anti-microbial activities of sAvBD1 ‘NYH’, ‘SSY’ and ‘NYY’ peptides

The amino acid changes underpinning the NYH, NYY and SSY AvBD1 variants did not significantly affect either the molecular weights (4645, 4671, 4568 Da) or net charges of the variants (+7.8, +7.7, +7.7). To investigate the potential effects of the amino acid changes on functionality the antimicrobial properties of synthetic NYH, SSY and NYY AvBD1 peptides were compared using bacterial strains isolated from the gastrointestinal tracts of commercially raised birds.

**Fig. 1:**
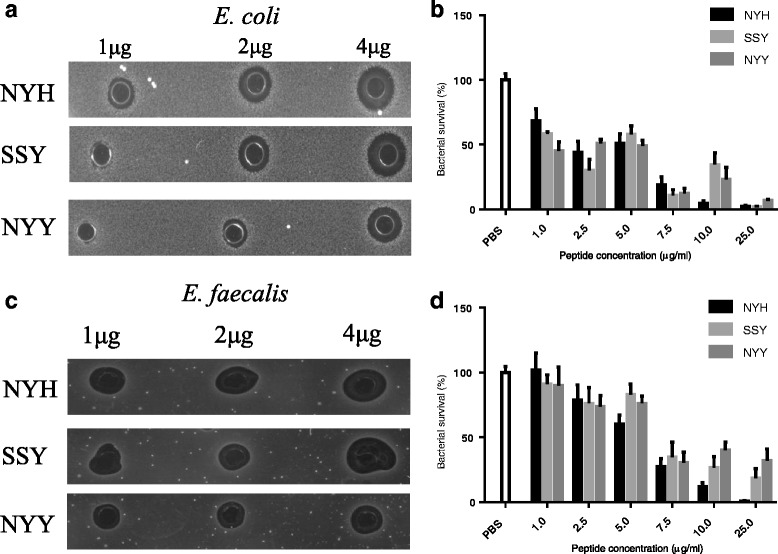
Antimicrobial Activities of the AvBD1 Variants (aerobic environment): Radial diffusion data showing inhibitory effects of AvBD1 variants against **a**) *E.coli* and **c**) *E.faecalis*. Time-kill assay data showing bacterial growth following 2 h incubation of **b**) *E.coli* and **d**) *E.faecalis* with AvBD1 variant peptides (Data presented as mean ± SEM; *N* = 3 experiments and minimum of 6 replicates)

The radial diffusion assay data, although qualitative, suggested a hierarchy of NYH > SSY > NYY against both Gram negative and positive bacteria isolated from the chicken GI tract (Fig. 1a, c). At 1. 2.5 and 5 μg/ml the time-kill assay data did not discriminate between the variants. However, at 10 μg/ml the ‘NYH’ form of the peptide, synthesized by the Line X birds, was associated with only 5% *E.coli* survival (95% killing activity), which compared to 34.5% and 23% survival (65.5% and 77% killing) respectively for the SSY and NYY peptides. At 25 μg/ml bacterial survival was <10% for all three peptides. The NYH > SSY > NYY killing pattern was observed for *Enterococcus faecalis* although the bacteria were more sensitive to lower concentrations of the NYH peptide with 40% killing at 5μg/ml compared to 17 and 24% for SSY and NYY (Fig. 1b, d). Additionally at 25μg/ml bacterial survival was still evident for the SSY and NYY peptides (18% and 32% respectively).

**Fig. 2:**
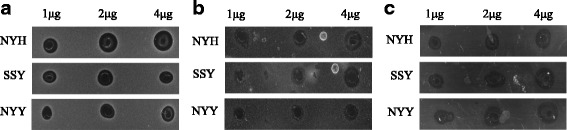
Antimicrobial Activities of the AvBD1 Variants (anaerobic environment): Radial diffusion data showing inhibitory effects of AvBD1 variant peptides against (**a**) *Lactobacillus johnsonii* (**b**) *Barnsiella viscericola* and (**c**) *Bacteroides dorei*

Radial diffusion assays performed under anaerobic conditions and used to explore the activities of the peptides against *Lactobacillus johnsonii* and *Barnsiella viscericola* (Fig. 2a,b) again supported the NYH > SSY > NYY antimicrobial hierarchy although the *Bacteroides dorei* data were less clear (Fig. 2c).

### Effects of AvBD1 on membrane Permeabilisation

**Fig. 3:**
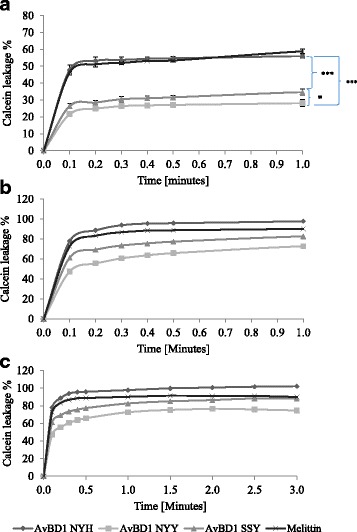
AvBD1 Variants and Membrane Permeabilisation: Calcein leakage from liposomes incubated with AvBD1 variant peptides 0.3uM (**a**) and 0.5uM (**b**) for up to 1 min, and 0.5uM up to 3 min (**c**). Experiments (*N* = 3;*n* = 3:mean ± SEM) performed at room temperature in 50 mM sodium phosphate buffer. *p < 0.05; ***p < 0.001

To further investigate the NYH > SSY > NYY antimicrobial hierarchy, the abilities of the three AvBD1 peptides to permeabilise bacterial membranes were explored using artificial liposomes loaded with calcein dye. The mean leakage assay data showed that sAvBD1 NYH, SSY and NYY at concentrations of 1.5 μg/ml (0.3 μM) induced 47.9%, 26.5% and 21.7% calcein leakage respectively, and within 6 s of addition (Fig. 3A). In each case leakage plateaued within three minutes at 58.9%, 38.6% and 32.7%, which compared to 70.5% for melittin, known for its permeabilisation properties [23]. Increasing AvBD1 to 2.5 μg/ml (0.5 μM) induced 78.0%, 61.4% and 47.5% leakage within 6 s, with leakage stabilised at 3 min (Fig3B&C) and recorded as 100% (NYH), 88.0% (SSY) and 74.6% (NYY) respectively. These data suggested that the sAvBD1 NYH peptide was the most potent membrane permeabilising agent and supported a peptide hierarchy of NYH > SSY > NYY.

### AvBD1 peptide secondary structure

**Fig. 4:**
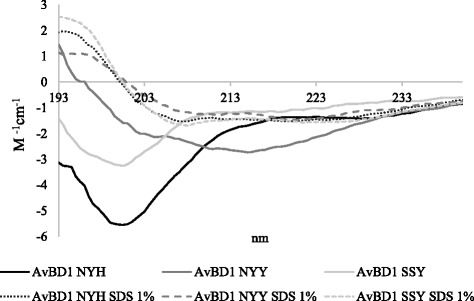
CD spectra of AvBD1 variant peptides. Peptides 250 μg/ml were analysed in 50 mM sodium phosphate buffer and following exposure to 1% SDS

Previous studies, focussed on ostrich defensins and NK-lysin, linked membrane leakage properties and bacterial killing to peptide charge [11, 24]. This could not be validated for the three AvBD1 peptides as their net charges were comparable. UV circular dichroism was used to explore whether the AMA and permeabilisation data could be explained by changes in the secondary structure conformations of the peptides following contact with the bacterial lipid membranes. The CD spectra of the linear NYH, NYY and SSY peptides in aqueous solution (10 mM phosphate buffer solution) predicted mostly unordered structure, but with some α-helix and β-sheet content (Fig. 4). Following exposure of the peptides to 1% SDS to model the anionic environment of the bacterial membrane, an increase in α-helix content was observed for all three peptides, although no significant differences in secondary structure were identified.

### AVBD1 modelling

**Fig. 5:**
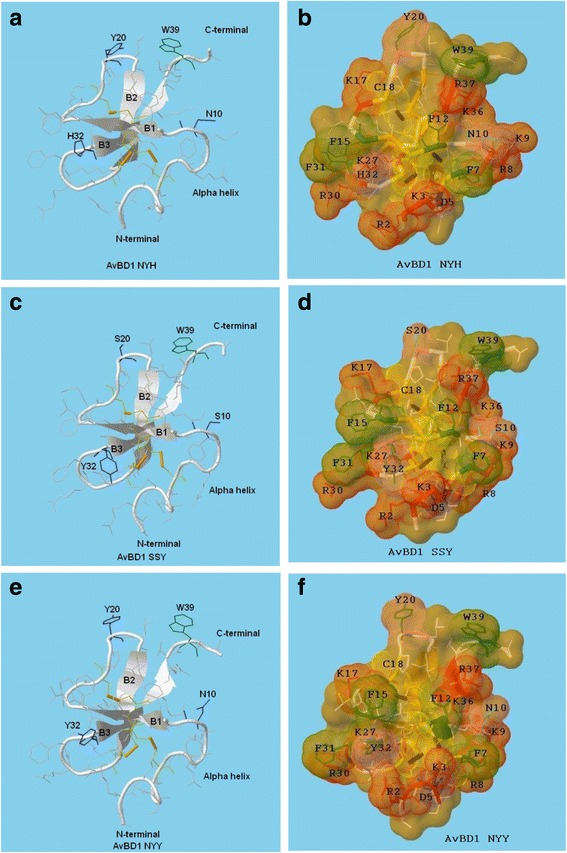
Predicted secondary structures and simulated 3D structures of AvBD1 variants NYH (**a**/**b**), SSY (**c**/**d**) and NYY (**e**/**f**)

To further investigate reasons for the differences in antimicrobial activities in silico modelling of the three variant sequences was used. The models predicted folded structures all containing comparable backbones displaying significant β-sheet content linked to three di-sulphide bonds and an N-terminal α-helix. All models indicated externalisation and clustering of positively charged and hydrophobic side chains including those of four phenylalanines, F7, F12, F15 and F31. The 3D model of AvBD1 NYH appeared unique with the side chains of W39/K9 and R30/2 associated with ‘claw-like’ structures; the former structure also linked to the positive charges of lysine K36 and arginine R37, and the latter to lysines K27 and K3, and histidine H32 (Fig. 5A/B).

Although the SSY model predicted a similar configuration to NYH, subtle changes were evident. The replacement of Y20 with a negatively charged serine residue disrupted the hydrophobicity of the Y20/W39 cluster, and this was associated with the lessening of the claw-like structure (Fig. 5C/D). The model relating to the NYY variant was characterised by the attenuation of the two claw-like structures, presumably a direct consequence of the N10, Y20 and Y32 side chains impacting on peptide folding (Fig. 5E/F).

### Bird gut Microbiotae

**Fig. 6:**
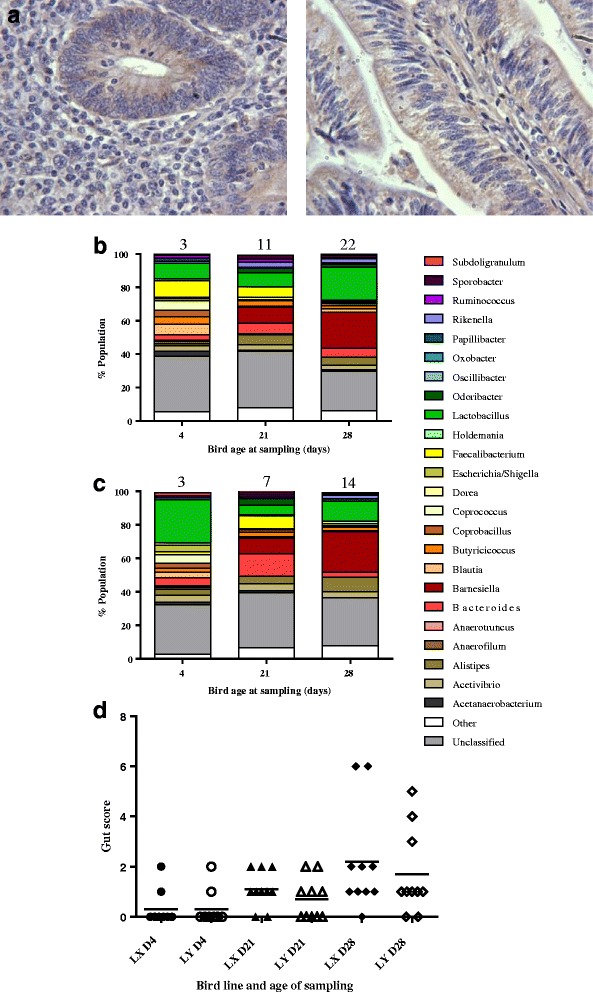
AvBD1 IHC and caecal microbiota analyses of LX and LY birds. IHC analyses to show epithelial localisation of AvBD1 in caecal tissues of Day 4 LX birds using AvBD1 polyclonal antibody diluted 1:250 and peroxidase staining (×400 magnification) (**a**). Caecal microbiota profiles of LX (**b**) and LY (**c**) birds (two independent trials using two different hatches) presented as relative abundance of bacterial genus (% microbial population). Corresponding total gut health scores/sampled group at each time point is shown above each column. (**d**) individual gut health data focussing on redness, watery digesta and gut tone where 0 (normal), 1 (some abnormalities) or 2 (very abnormal), and poorest guts scoring 6

Caecal gut epithelial synthesis of AvBD1 was confirmed by IHC (Fig. 6A). Analyses of the microbiotae residing in the caecae of LX and LY birds, at ages 4, 21 and 28 days are presented in Fig. 6B & C. These data relate to 60 birds sampled from two independent trials involving two different hatches. Of the genera identified *Lactobacilli sp.* were predominant in the caeca of both lines, LX and LY, at the three sampled time points. While these *Lactobacilli sp.* data suggested trends at Days 4 (9% LX vs × 30% LY; *P* = 0.394) and 28 (20% LX vs 12%LY; *P* = 0.323) respectively, no statistically significant changes were detected. The emergence and colonisation of the caeca with *Barnsiella sp.* were also evident in both lines at Days 14 (9.5% LX vs 9.3%LY) and 28 (22% LX vs 26% LY), and the patterns were consistent between the two lines. There were no significant differences in the gut scores of the two lines although the data suggested the LY birds to have healthier guts overall (Fig. 6C & D).

## Discussion

The earliest response of epithelia to microbial challenge is mediated through the synthesis of innate immune factors and the defensins are key innate elements that function to protect the host from potential pathogens as well as controlling the commensal microbial communities [25]. As genetic variation can potentially increase an organism’s susceptibility to infection and disease, defensin gene SNPs resulting in non-synonymous amino acid changes are rare. Analyses of commercial chicken broiler lines identified three lines carrying variants of the AvBD1 gene encoding non-synonymous amino acid changes at positions 10, 20 and 32 of the mature peptide. The resulting peptides were designated as AvBD1 NYH, SSY and NYY, and comparison of their killing activities indicated all to have antimicrobial properties against gram negative and positive bacteria associated with the chicken gut, but with a hierarchy of NYH > SSY > NYY.

Cationic AMPs, including the defensins, are perceived as targeting bacterial membranes through electrostatic interactions and facilitating killing through permeation of the intact membranes causing pore formation, and leakage of the intracellular content [26, 27]. While it is acknowledged that the in vitro AMA profiles of the three AvBD1 peptides reflected their linear forms, this scenario probably reflected the physiological situation in vivo where the anaerobic environment of the gut, particularly the colon, favours the reduced form of the peptide [15]. In vitro all three linear AvBD1 peptides showed transition to an α-helical structure following exposure to an anionic environment, which supported a classical pore-killing mechanism in which the α-helix lines up parallel to the membrane, inserts into the bilayer and disrupts membrane permeability, leading to microbial cell death [28]. However, using CD analyses the changes in peptide secondary structure between the peptide variants were similar and hence could not explain the consistent observation that AvBD1 NYH peptide was the most potent membrane permeabilising and killing agent. Linear AMPs have been shown to function directly through membrane pores [29], but the actual mechanisms can differ with some peptides including lactoferricin (Lfcin B) associated with local rupture events prior to pore formation [30]. Therefore in explaining the observed hierarchy of the AvBD1 molecules it cannot be excluded that the presence and positioning of the AvBD1 NYH, NYY and SYY amino acids supported novel rupture-like mechanisms.

Previous studies, focussed on ostrich defensins and avian NK-lysin, linked membrane leakage properties and hence microbial killing to peptide charge [11, 24]. This association was not validated for AvBD1 as the net charges of the three peptides were comparable. Similar observations challenging the charge and killing potency model were also reported for Apl_AvBD2 and AvBD8, where the substitution of residues that impacted on peptide charge (F/R and I/R) did not improve antimicrobial activity [31, 32]. Moreover, the in vitro modelling of a SNP encoding a non-synonymous R/I substitution identified in the Great Tit AvBD7 gene, indicated that only the isoleucine allelic form associated with the lower charged peptide (+3.7 v + 4.7), was associated with the inhibition of *Staphylococci* growth [33]. So while cationic charge is important for peptide killing other factors yet to be determined appear to be involved in the AvBD1 mode of action. For example it could not be excluded that the presence of H32 increased the antimicrobial capacity of the AvBD1 NYH variant through localised charge effects.

Modelling of the AvBD1 molecules indicated their native folded structures to be comparable, with each displaying a three-stranded antiparallel β-sheet structure. In contrast to AvBD2 [34], they were all predicted a short N-terminal helical segment, the importance of which in bacterial killing, was supported by the CD spectra data. All three molecules were projected to display externalisation of positively charged and hydrophobic residues with the localisation of positively charged amino acids R30, K27, R2 and K3, lending support to a strong electrostatic interaction of the defensin molecules with anionic bacterial membranes. Additionally F15, F31 and F7 provided interfacial hydrophobicity, with phenylalanine residues shown previously by NMR to play key roles in the binding of a cationic peptides to a negatively charged membrane [35]. Structural modelling also identified distinct claw-like regions, which have been suggested previously to play a key role in microbial membrane attachment [32]. The claw regions were more distinct in the NYH model and it is feasible in vivo that such structures are involved and key factors in its membrane attachment and permeating abilities.

Bioinformatics analyses of the AvBDs highlighted a number of positively selected sites that would mutate at a higher rate than expected [36, 37]. For AvBD1 these corresponded to SNP2 (Y/S) and SNP3 (Y/H), and the data presented herein supports these predictions. Moreover, while indicating a definite hierarchy, these data also suggested that the amino acids encoded by the SNPS are not detrimental to peptide killing and hence mucosal host immunity, which probably explains their selection, tolerance and conservation within the broiler chicken lines. Gut performance is unlikely to rely on or reflect the activity of a single gut antimicrobial peptide, but it is known that caecal AvBD1 expression is maximal immediately post hatch and maintained up to day 7 [13], hence the increased potency of NYH variant could impact on the composition of the caecal gut microbiota.

*Lactobacilli sp.* have been reported to be important for immune development of the gut [38], with abundant *Lactobacilli* numbers linked to a robust bird gut immune system [39]. In vitro*,* the NYH peptide proved the most potent form against *L. johnsonii,* a recognised gut probiotic [40]. This raised the possibility that its synthesis by the young LX birds could impact their gut health through reduced colonisation with *Lactobacilli*, and exposure of the gut epithelia to other potentially less protective bacterial species. While the caecal microbiota data suggested the reduced abundance of *Lactobacilli* in the microbiota of the Day 4 LX compared to LY birds, these data were not statistically significant and hence did not support the peptide as playing a role in gut health.

The observations supporting the in vivo synthesis of three AvBD1 peptides with different antimicrobial activities appear to conflict with the chicken NK-lysin protein where the most potent antimicrobial form is favoured genetically [12]. However, unlike the defensins that are synthesised by epithelial cells and heterophils [41], NK-lysin molecules are synthesised and stored in the cytotoxic granules of T lymphocytes, and Natural Killer cells. Their release is part of a cell-mediated immune response that has evolved and functions to destroy microbes, particularly those surviving intracellularly. Hence the necessity for intracellular pathogen killing has presumably driven the selection of the most potent NK-lysin agent.

## Conclusion﻿s

This study identified three non-synonymous SNPs in the AvBD1 gene encoding the variant peptides NYH, SSY and S/NYY that segregated into three lines of commercial broiler chickens LX, LY and LZ. While the peptide antimicrobial hierarchy was determined in vitro as NYH > SSY > NYY these changes did not appear to impact on bird gut microbiota or health. However, these data highlight the need for breeders to consider the impact of host defence molecules on the commensal as well as pathogenic populations in young birds as simply selecting for the most potent version of genes encoding peptides playing roles in innate immunity may not be beneficial commercially.

## Abbreviations

AMA: Antimicrobial activity
AvBD: Avian β-defensin
AvBD1: Avian β-defensin-1
IHC: Immunohistochemistry
NYH: Asparagine, tyrosine, histidine
NYY: Asparagine, tyrosine, tyrosine
SNP: Single nucleotide polymorphism
SSY: Serine, serine, tyrosine
